# Integrative computational analysis of public fecal lipidomics and transcriptomics datasets suggests a candidate association between the COX-2 pathway and CE(20:4) in colorectal adenoma-carcinoma progression

**DOI:** 10.1371/journal.pone.0358259

**Published:** 2026-09-25

**Authors:** Bing Tang, Yanru Chen, Xia Yang, Hanlin Gong

**Affiliations:** 1 Division of Surgery, Institute of Integrated Traditional Chinese and Western Medicine, West China Hospital, Sichuan University, Chengdu, China; 2 Department of Rehabilitation, The People’s Hospital of Jianyang City, Jianyang, China; University of Bonn, Institute of Experimental Hematology and Transfusion Medicine, GERMANY

## Abstract

**Background:**

The fecal lipidomic changes underlying the colorectal adenoma-carcinoma sequence remain incompletely characterized, particularly regarding the continuous metabolic trajectory and heterogeneity of precancerous adenomas. We aimed to use publicly available multi-omics data and an integrative computational framework to identify candidate lipidomic features associated with a CRC-like metabolic state in adenomas.

**Methods:**

We developed an extreme-phenotype machine learning strategy using fecal lipidomics from healthy controls and colorectal cancer (CRC) patients (Study ID: ST003798) to build a diagnostic model, which was then blindly applied to adenoma patients to compute a Fecal Lipidomic Malignancy Risk Score (FL-MRS), interpreted here as a CRC-like lipidomic similarity score. SHAP analysis prioritized influential lipid features, and cross-sectional pseudotime trajectory inference reconstructed the metabolic continuum. Transcriptomic data from TCGA-COAD and single-cell RNA-seq (Broad Institute) were integrated to explore potential tissue-level correlates. Targeted single-molecule trend verification of the top lipid candidate was performed in an independent cohort (ST002787), as full model replication was not feasible due to limited inter-cohort feature overlap. Multiple sensitivity analyses were conducted to assess the robustness of the computational pipeline.

**Results:**

The Random Forest model showed robust performance on the independent test set (AUC = 0.864). When applied to adenomas, 51.7% of patients exceeded the FL-MRS threshold derived from extreme phenotypes; however, this proportion far exceeds the known clinical adenoma-carcinoma progression rate (approximately 5–10%), indicating that FL-MRS should be interpreted as a metabolic similarity metric rather than a direct cancer risk probability. SHAP analysis prioritized arachidonic acid-derived cholesterol ester CE(20:4) as the top predictive feature, with 100% bootstrap selection frequency. Integrative analysis of independent transcriptomic datasets identified upregulation of *PTGS2* (COX-2) in tumor tissue and its predominant expression in stromal cells. External single-molecule targeted verification confirmed an accumulation trend of CE(20:4) and an early adenoma-phase peak of eicosanoid mediators (including oxidative stress and LOX-pathway metabolites). Notably, CE(20:4) levels did not differ significantly between adenoma and CRC (*P* = 0.055), consistent with its proposed role as an early event marker. Pseudotime trajectory inference was insensitive to root node assignment.

**Conclusions:**

This computational study suggests that fecal CE(20:4) and the associated COX-2 pathway may represent candidate features of a CRC-like metabolic state in colorectal adenomas. FL-MRS is not proposed as a clinical diagnostic or risk-prediction tool; rather, it serves as a research instrument for quantifying CRC-like metabolic similarity and guiding future validation studies. All findings are derived from publicly available retrospective data without independent experimental validation and should be regarded as hypothesis-generating. The complete analysis code is publicly archived to ensure reproducibility and facilitate future validation.

## Introduction

The accumulation of high-throughput omics data in public repositories has created unprecedented opportunities for biomarker discovery and disease mechanism research [1–3]. However, extracting continuous disease progression trajectories from cross-sectional data and transparently translating computational model outputs into testable biological hypotheses remain significant methodological challenges. Reusing existing datasets with rigorous analytical frameworks offers a cost-effective strategy to generate such hypotheses, provided that the limitations of retrospective, unpaired data are explicitly acknowledged.

Colorectal cancer (CRC) develops through the well-established adenoma-carcinoma sequence [4–8], providing an ideal disease model for studying stepwise metabolic reprogramming. Fecal lipidomics has emerged as a non-invasive approach that reflects host-microbiome metabolic interactions [9–11]. Previous studies have primarily focused on distinguishing CRC from healthy controls [12–15], often treating adenomas as a homogeneous intermediate category or excluding them to avoid transitional noise [16,17]. Although some recent work has begun to explore metabolic heterogeneity within adenomas, the continuous lipidomic trajectory from healthy mucosa through adenoma to CRC remains incompletely mapped using computational approaches. The extreme-phenotype machine learning strategy, which trains classifiers exclusively on clinical extremes to probe intermediate states, has been applied in other disease contexts and omics domains, but its application to fecal lipidomics and colorectal adenoma-carcinoma progression has not been systematically evaluated.

To address this gap, we developed an extreme-phenotype machine learning framework. This strategy trains a classifier exclusively on the two clinical extremes—healthy controls and overt CRC—intentionally omitting adenomas from model building. The trained model is then used to compute a continuous Fecal Lipidomic Malignancy Risk Score (FL-MRS) for unseen adenoma samples. We combined this approach with SHapley Additive exPlanations (SHAP) for model interpretation [18–20] and cross-sectional pseudotime inference [21–23] to prioritize candidate lipid features and reconstruct the metabolic landscape. To explore potential tissue-level correlates, we integrated TCGA-COAD bulk transcriptomics [11] and single-cell RNA sequencing (scRNA-seq) data from the Broad Institute [6]. An independent fecal lipidomics cohort (ST002787) was used for targeted single-molecule trend verification of the top candidate molecule. Furthermore, we conducted multiple sensitivity analyses—including alternative missing value imputation strategies, pseudotime root node reversal, and bootstrap-based feature selection stability assessment—to comprehensively evaluate the robustness of our computational pipeline. While individual components of this framework have been used in other contexts, their integration for probing the adenoma-carcinoma metabolic continuum in fecal lipidomics represents the specific contribution of this study.

It is critical to emphasize that this study is entirely a computational re-analysis of publicly available datasets. No new experimental data were generated, and all multi-omic associations are derived from independent, unpaired cohorts. Consequently, our findings should be viewed as hypothesis-generating and do not establish causality. We have made all analysis code publicly available to ensure full transparency and to facilitate independent verification of our results. The present study is positioned as a hypothesis-generating computational investigation, distinct from traditional experimental biomarker discovery research.

## Materials and methods

### Study design and cohort description

We performed a retrospective, multi-cohort computational analysis. The discovery fecal lipidomic cohort (Study ID: ST003798) comprised healthy controls (*n* = 78), CRC patients (*n* = 75), and adenoma patients (*n* = 58) [24]. An independent targeted verification cohort (ST002787) included healthy controls (*n* = 30), adenomas (*n* = 37), and CRC (*n* = 35) [25]. Tissue-level transcriptomic data were obtained from TCGA-COAD (tumors *n* = 481, normal *n* = 41) [26], and scRNA-seq data from the Broad Institute (c295) [27]. Of note, key clinical covariates (age, sex, BMI, medication use, dietary habits, and adenoma histopathological grading) were not available in these public datasets; this limitation is addressed in the Discussion. All datasets were accessed for research purposes between April 1, 2026 and June 1, 2026. The authors did not have access to any information that could identify individual participants during or after data collection.

This study is a secondary analysis of de-identified, publicly available data. The original collections received ethics committee approval, and all subjects provided written informed consent. Additional institutional review board approval was waived for this secondary analysis. The study conformed to the Declaration of Helsinki. A schematic overview of the multi-cohort study design is provided in S2 Fig in S1 File.

### Extreme phenotype machine learning framework

Raw lipidomic matrices underwent quality control, TIC normalization, KNN imputation (*k* = 10), and $\log_{2}$ transformation. The adenoma group was entirely sequestered as a blinded evaluation set. Using only healthy controls and CRC patients, we split the data into training (70%) and independent test (30%) sets, stratified by clinical outcome. Feature selection was performed via LASSO with 10-fold cross-validation, adopting the strict *lambda.1se* criterion to minimize overfitting. A Random Forest classifier (*ntree* = 500) was built on the selected signature. The number of trees was confirmed by visual inspection of the out-of-bag (OOB) error stabilization plot to be sufficient for convergence (S3 Fig in S1 File). The FL-MRS cutoff was defined using Youden’s index derived from internal cross-validation and subsequently applied blind to the test set and the adenoma cohort. A detailed justification for selecting Random Forest over other classifiers is provided in S5 Table in S2 File. In addition to the single 7:3 split, we report the median AUC and 95% CI from 1,000 stratified bootstrap resamples to assess split-to-split variability.

### Sensitivity analysis of missing value imputation

To assess whether the choice of imputation strategy affected feature selection, we conducted a sensitivity analysis using an alternative LOD/2 (half-minimum) imputation approach without prior feature removal. The missing values in the raw data are primarily left-censored (below detection limit), for which KNN and LOD/2 imputation rest on fundamentally different assumptions. The full LASSO pipeline was reapplied to this LOD/2-imputed matrix. Feature overlap with the original 11-feature signature was quantified using Jaccard similarity. Furthermore, we performed 100-iteration bootstrap LASSO stability analysis to assess the selection frequency of each feature under repeated subsampling of the training data. Detailed results are provided in S7 and S11 Tables in S2 File.

### Explainable AI and pseudotime trajectory inference

SHAP values were computed via Monte Carlo sampling (100 simulations) to interpret feature contributions [28]. To reconstruct the metabolic landscape from cross-sectional data, we performed PCA on centered and scaled lipidomic data and applied principal curve analysis (*princurve* package) based on the first two principal components. The first two principal components were selected to enable two-dimensional visualization required by the principal curve algorithm; this dimensionality reduction may omit metabolomic information captured in higher-order components, and the trajectory results should therefore be interpreted as exploratory. The healthy control group was designated as the root. We emphasize that this pseudotime inference is a computational ordering derived from static measurements and does not represent true longitudinal progression. To assess the sensitivity of the pseudotime ordering to root node selection, we repeated the analysis with the CRC group assigned as the root and compared the relative ordering of high-risk versus low-risk adenomas (S4 Fig in S1 File).

### Targeted external verification

Due to the limited overlap of detected lipid features between the discovery (ST003798) and external verification (ST002787) cohorts—only 1 of the 11 model features was detected in both datasets (S6 Table in S2 File)—full model replication was not technically feasible. Instead, we performed a targeted single-molecule trend verification of the top-ranked lipid, CE(20:4), and two eicosanoid mediators associated with arachidonic acid metabolism (8-iso Prostaglandin E2 and Tetranor-12(R)-HETE) across the healthy-adenoma-carcinoma continuum. Between-group differences were assessed using the Kruskal-Wallis test with Dunn’s post-hoc test and Benjamini-Hochberg correction (S8 and S9 Tables in S2 File). Of note, 8-iso Prostaglandin E2 is an isoprostane formed via non-enzymatic lipid peroxidation and is not a direct COX-2 enzymatic product; it serves here as a marker of oxidative stress, which is known to be associated with COX-2 induction in the colorectal mucosa. Tetranor-12(R)-HETE is a metabolite of 12-HETE, primarily generated via the lipoxygenase (LOX) pathway, and should not be interpreted as a COX-2-specific metabolite.

### Transcriptomic and single-cell data integration

Differential expression of *PTGS2*, *SOAT1*, and *PLA2G4A* between normal (*n* = 41) and tumor (*n* = 481) tissues in the TCGA-COAD cohort was assessed using Welch’s t-test. Although the Shapiro-Wilk test indicated deviation from normality for all three genes in tumor tissue (S1 Table in S2 File), Welch’s t-test is robust to such deviations given the large sample size. This was further confirmed by Wilcoxon rank-sum test, which yielded consistent results (*P* = 0.0032 for *PTGS2*; S10 Table in S2 File). scRNA-seq data were processed with Seurat (v4.4.0). We stress that these transcriptomic datasets originate from cohorts completely independent of the fecal lipidomic discovery cohort; therefore, all identified associations are *in silico* inferences.

### Statistical analysis and software

Analyses were conducted in R v4.3.1. Internal validation of the FL-MRS was performed with 1,000 stratified bootstrap resamples. All code is publicly archived at https://github.com/bingmoon/FL-MRS_Trajectory. This study is reported in accordance with the TRIPOD reporting checklist (S3 File). Key methodological constraints and limitations of this computational framework are extensively discussed in the Discussion section.

## Results

### Extreme phenotype modeling defines a CRC-associated lipidomic signature

**Fig 1:**
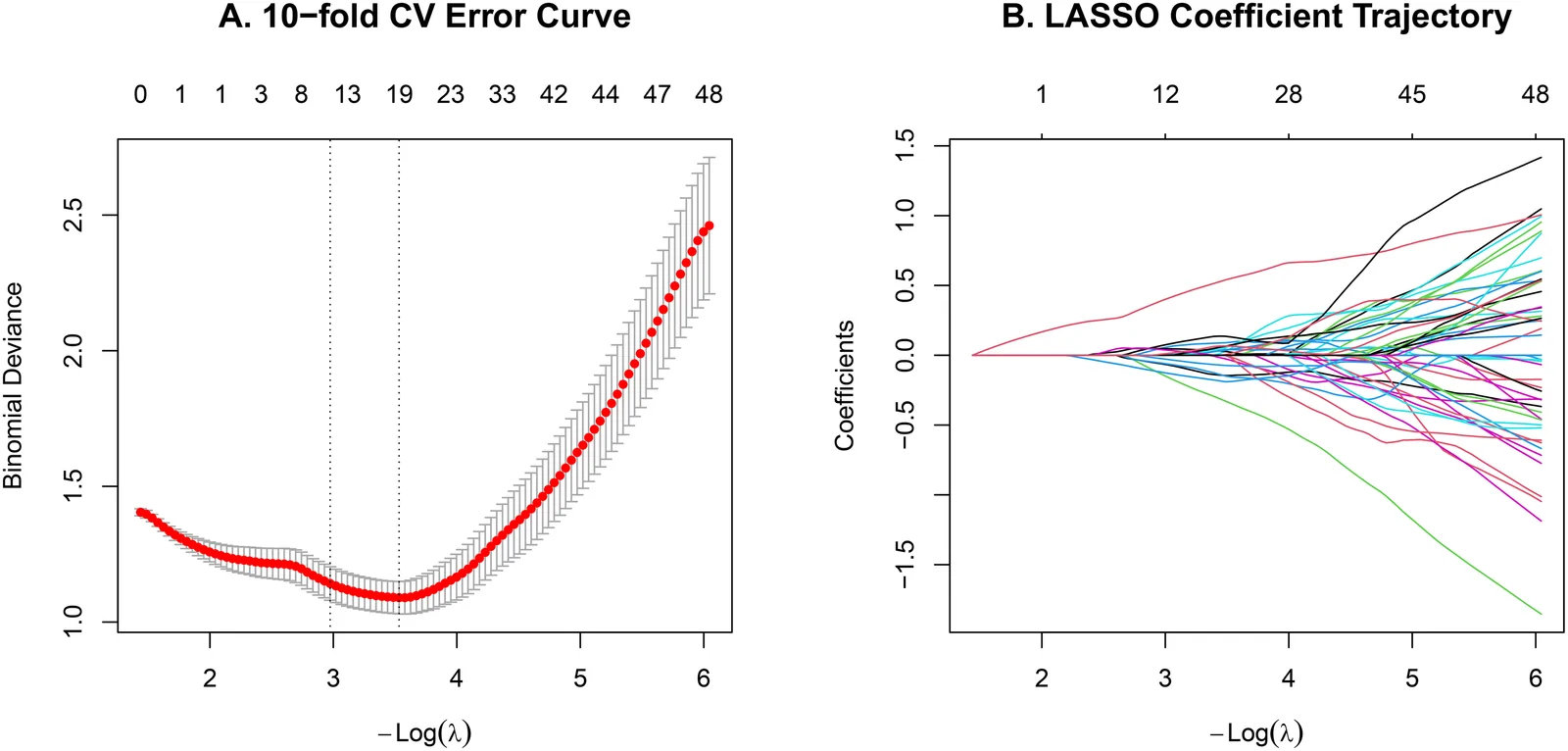
Feature selection via LASSO regression (ST003798 cohort). (A) 10-fold cross-validation error curve showing the selected optimal λ (lambda.1se, the largest λ within one standard error of the minimum cross-validation error). (B) Coefficient trajectory for fecal lipid metabolites. Each colored line represents one lipid feature; features with non-zero coefficients at lambda.1se were selected for the final model. The complete list of selected features is available in S4 Table in S2 File.

**Fig 2:**
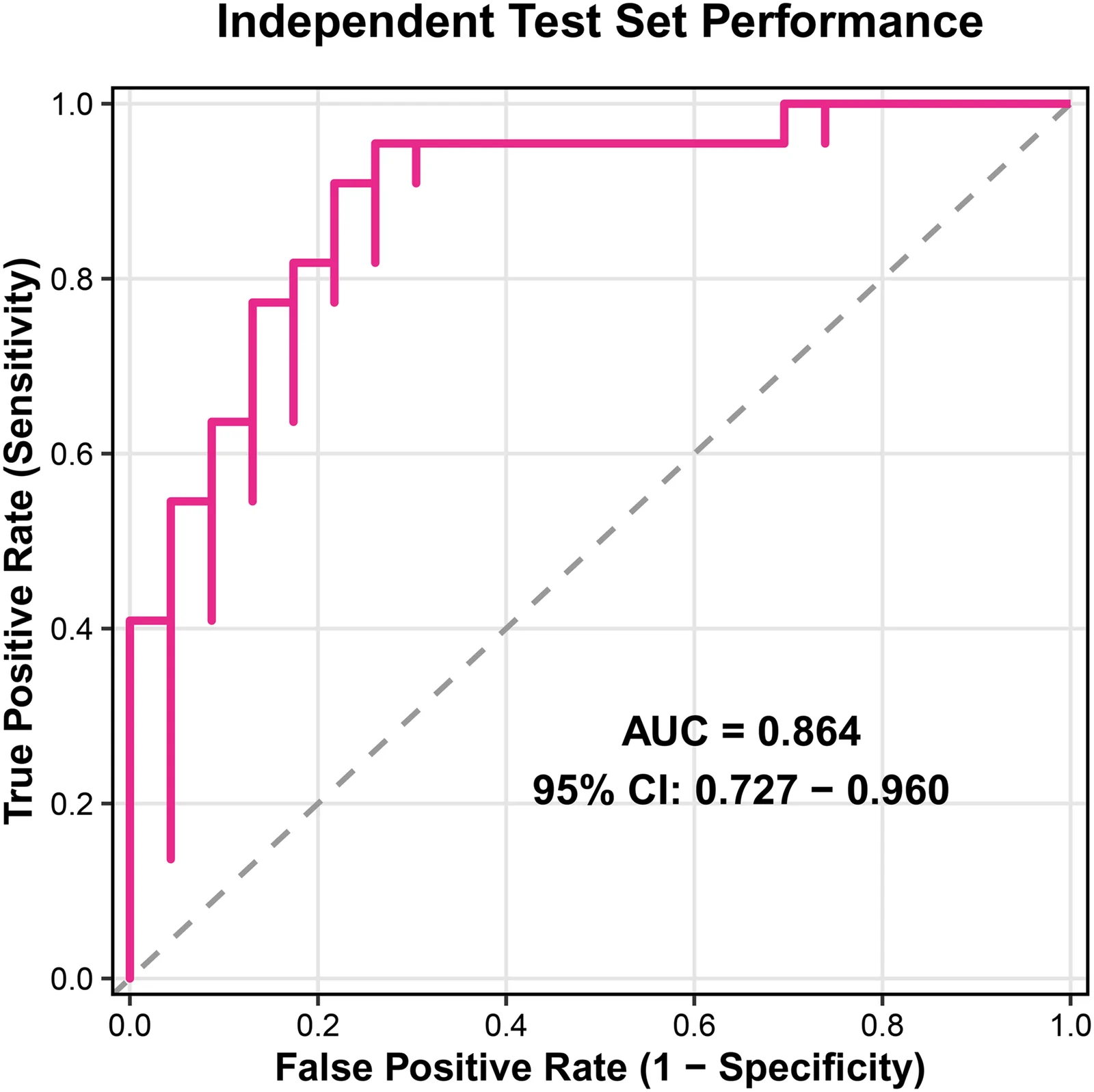
Receiver operating characteristic (ROC) curve of the Random Forest model on the independent test set (*n* = 45). AUC, area under the ROC curve (0.864; 95% CI: 0.727–0.960). The diagonal dashed line represents the performance of a random classifier (AUC = 0.5).

LASSO regression identified 11 core lipid features distinguishing healthy controls from CRC (S4 Table in S2 File and Fig 1). The Random Forest model achieved an AUC of 0.864 (95% CI: 0.727–0.960) with an accuracy of 82.2%, sensitivity of 95.5%, specificity of 68.8%, positive predictive value of 75.0%, and negative predictive value of 94.1% on the independent test set (Fig 2; full diagnostic metrics in S3 Table in S2 File). Notably, the model showed relatively lower specificity (68.8%), reflecting the inherent complexity of fecal lipidomic classification and the asymmetric penalty for false positives versus false negatives in cancer screening contexts. The OOB error stabilized at 416 trees with a minimum error rate of 0.1944, confirming that 500 trees were sufficient for convergence (S3 Fig in S1 File). Internal bootstrap validation yielded a median AUC of 0.862 (95% CI: 0.727–0.960), confirming the stability of the chosen cutoff (0.446). The model was well-calibrated and offered superior net clinical benefit across a range of thresholds (S1 Fig in S1 File).

### FL-MRS reveals considerable heterogeneity among adenomas

**Fig 3:**
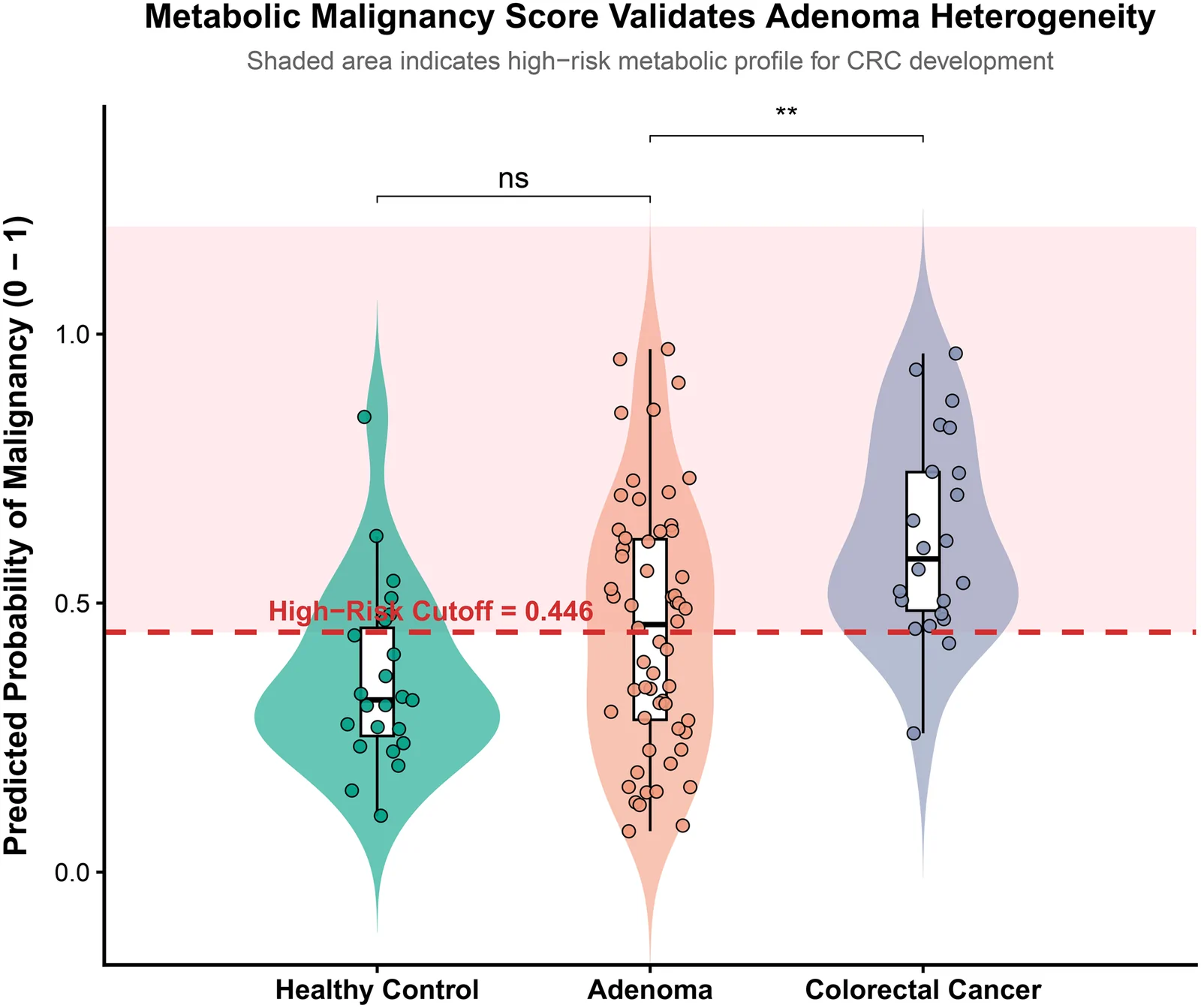
Fecal Lipidomic Malignancy Risk Score (FL-MRS) unmasks the hidden heterogeneity of colorectal adenomas. A blind assessment of adenoma samples (*n* = 58) reveals substantial metabolic diversity, with 51.7% exceeding the high-risk threshold (Cutoff = 0.446, derived from Youden’s index in the training set). The 51.7% proportion indicates substantial metabolic heterogeneity rather than direct progression risk; it far exceeds the known clinical adenoma-carcinoma progression rate (approximately 5–10%), confirming that FL-MRS should be interpreted as a CRC-like lipidomic similarity score, not a cancer risk probability.

Blind application of the FL-MRS to the adenoma cohort (*n* = 58) exposed substantial metabolic diversity: 51.7% (*n* = 30) exceeded the high-risk threshold of 0.446 (Fig 3). This proportion is substantially higher than the clinically observed adenoma-carcinoma progression rate (approximately 5–10% for advanced adenomas over 10 years), indicating that FL-MRS should not be interpreted as a direct cancer risk probability. Rather, it serves as a CRC-like lipidomic similarity score that reflects the degree of metabolic deviation from healthy controls toward a CRC-like state. However, in the absence of detailed histopathological grading and clinical covariates (e.g., inflammation, polyp size, NSAID use), we cannot determine whether this subgroup represents biologically more aggressive lesions or merely reflects unmeasured confounders. This observation therefore warrants cautious interpretation.

### SHAP analysis prioritizes CE(20:4) as a top predictive feature

**Fig 4:**
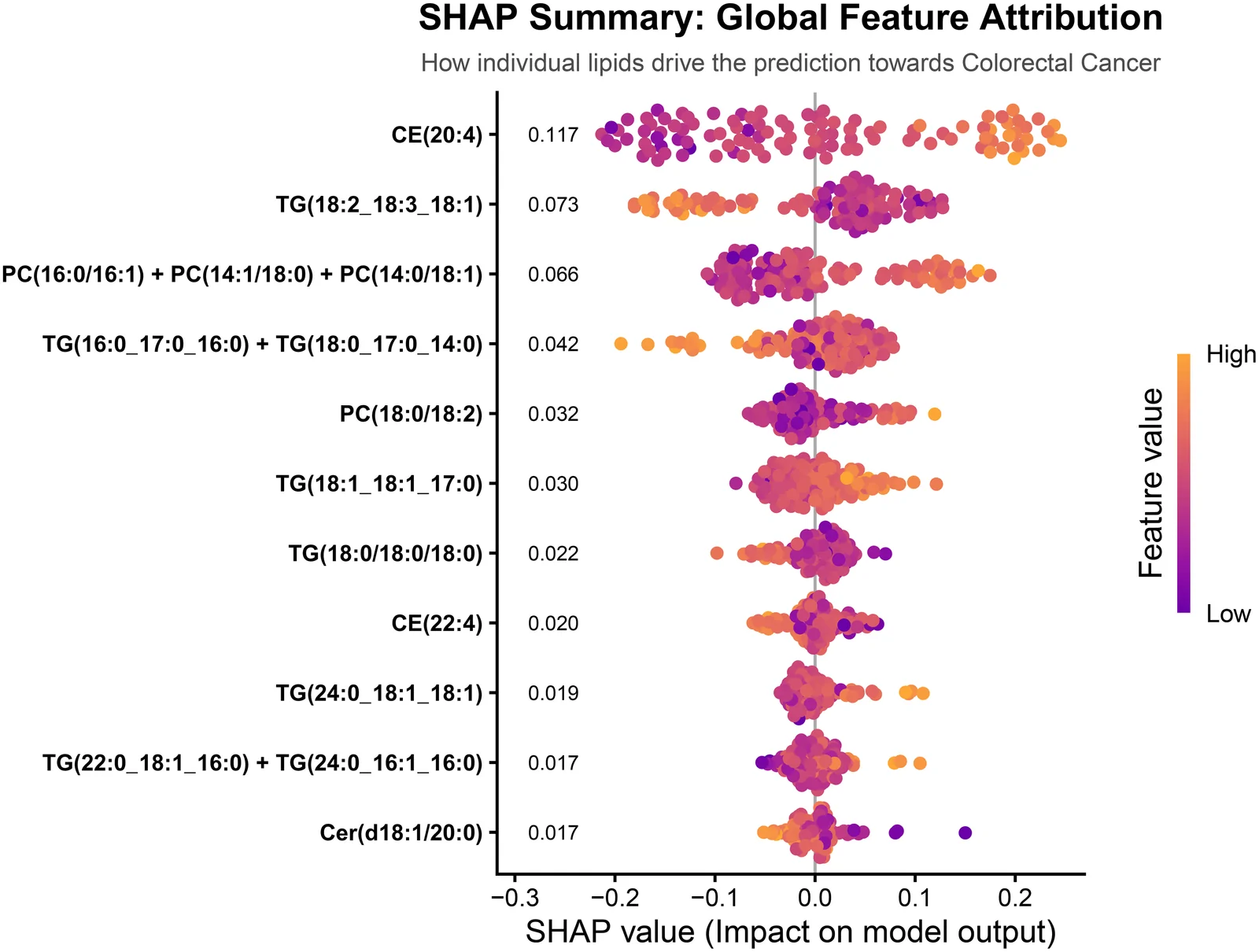
SHAP (SHapley Additive exPlanations) summary plot illustrating global feature attribution. Each point represents the SHAP value for one sample. Features are ranked by mean absolute SHAP value (importance). Color indicates feature value (red: high; blue: low). Positive SHAP values (right of zero) drive the model toward CRC prediction; negative values drive toward healthy prediction. CE(20:4) is the most influential feature.

**Fig 5:**
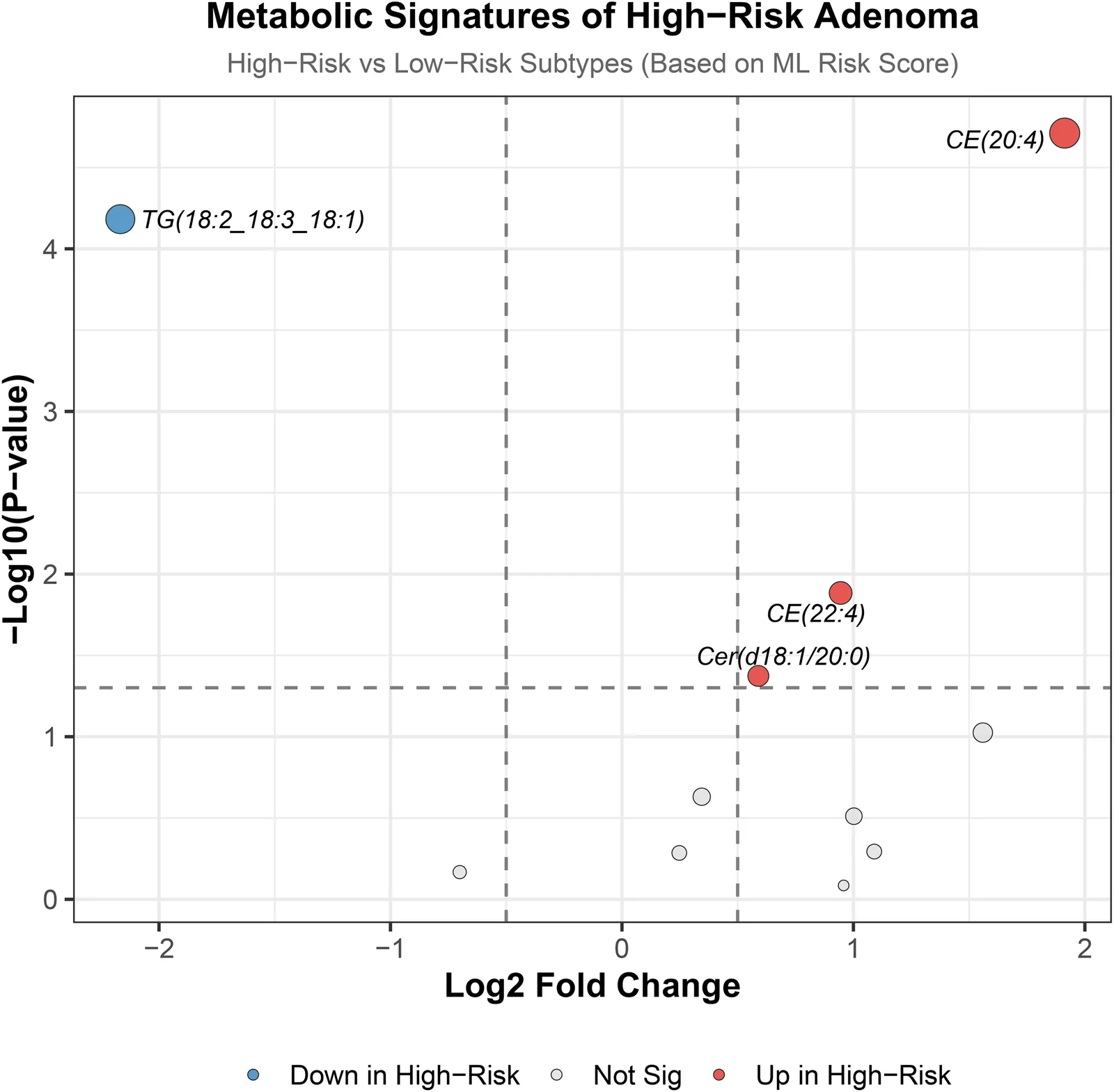
Volcano plot of metabolic differences between High-Risk and Low-Risk adenomas as defined by FL-MRS. Each point represents one lipid feature. The x-axis shows log2 fold change (positive values indicate higher abundance in the High-Risk group). The y-axis shows $-\log_{10}$(FDR-adjusted P-value) from Wilcoxon rank-sum tests. Dashed horizontal line: FDR < 0.0001; dashed vertical lines: |Log_2_FC| > 1. CE(20:4) is highlighted. This analysis serves as an internal model weight validation, as FL-MRS is itself constructed from these features.

Global SHAP analysis indicated that CE(20:4) was the most influential feature driving CRC-positive predictions, while certain polyunsaturated triglycerides were negatively associated with CRC risk (Fig 4). Sensitivity analysis using an alternative LOD/2 imputation strategy confirmed that CE(20:4) was the only feature consistently selected across both preprocessing pipelines (Jaccard similarity = 0.059 between the two feature sets; S7 Table in S2 File). Bootstrap LASSO stability analysis further demonstrated that CE(20:4) had a 100% selection frequency across 100 resampling iterations, while the remaining features showed variable stability (selection frequencies ranging from 36% to 95%; S11 Table in S2 File). This supports CE(20:4) as a robust candidate feature across multiple analytical pipelines, whereas the broader 11-feature signature should be interpreted with appropriate caution. The volcano plot comparing high-risk and low-risk adenomas (defined by FL-MRS) confirmed significant upregulation of CE(20:4) in the high-risk subgroup (FDR-corrected *P* < 0.001, $\log_{2}\text{FC}=1.914$, Fig 5). It should be noted that this comparison serves as an internal model weight validation rather than independent biological evidence, as FL-MRS is itself constructed from these features.

### Cross-sectional pseudotime analysis reconstructs a putative metabolic continuum

**Fig 6:**
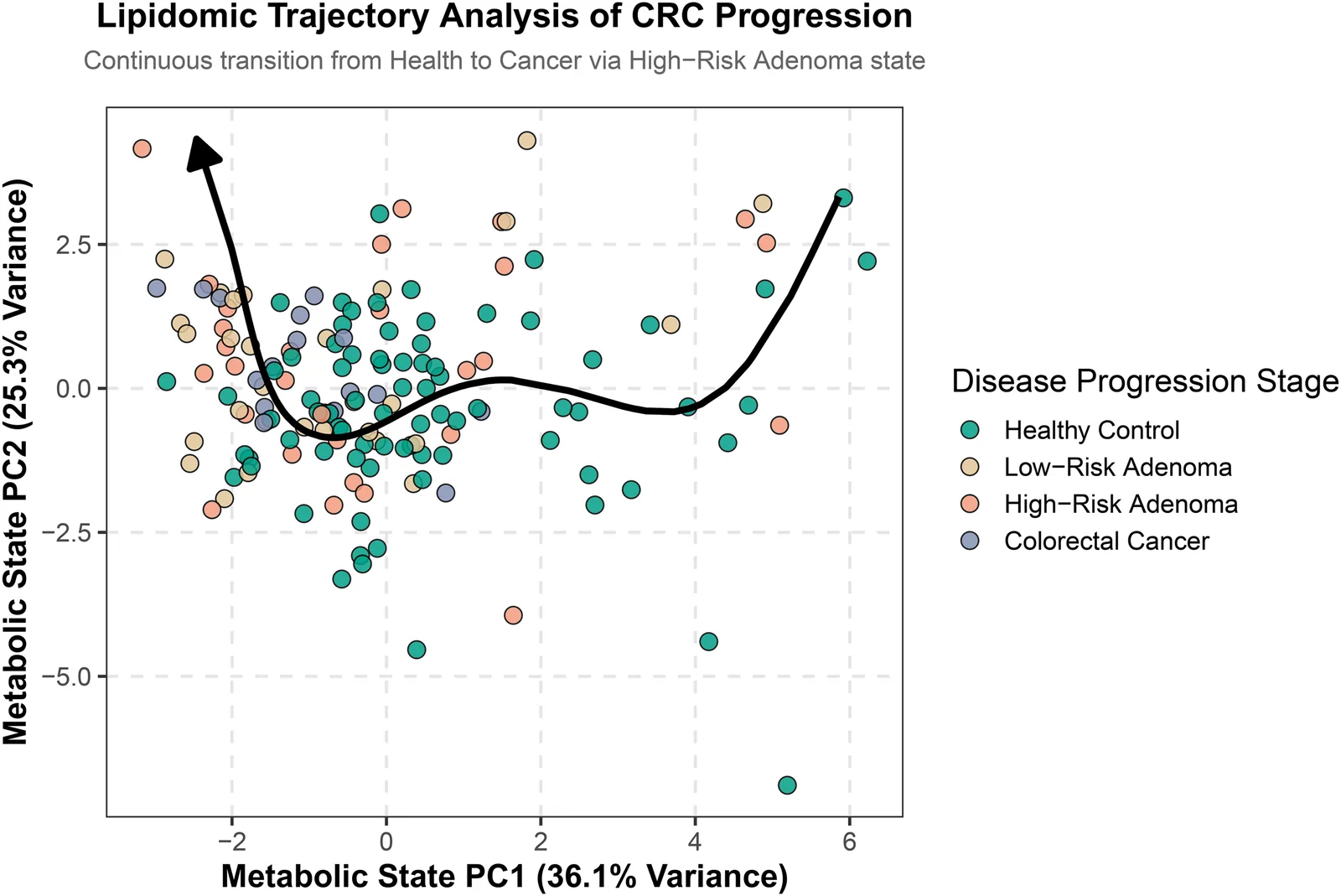
Pseudotime trajectory analysis mapping the continuous metabolic transition from healthy controls to CRC. Principal curve analysis based on the first two principal components (PC1 and PC2) of the lipidomic data (ST003798 cohort, total *n* = 211). Each point represents one sample. The curve represents the inferred pseudotime trajectory; λ denotes the arc-length (pseudotime) along this curve. Healthy controls were designated as the trajectory root (λ≈0). High-risk adenomas (red) show a visual trend toward the CRC terminus, though the group difference is not statistically significant (*P* = 0.66).

Principal curve analysis ordered samples along a continuous metabolic spectrum from healthy to CRC (Fig 6). A visual trend was observed wherein high-risk adenomas tended to localize toward the CRC terminus; however, this difference did not reach statistical significance (Wilcoxon rank-sum test, *P* = 0.66 for both root assignments; S4 Fig in S1 File). Pseudotime, as an unsupervised dimension derived from global lipidomic variation, is not expected to recapitulate the supervised FL-MRS classification, and the two metrics should be regarded as capturing complementary rather than mutually validating information. The modest correlation between pseudotime and FL-MRS (Spearman’s R = −0.128, *P* = 0.20) further supports this interpretation. Pseudotime root node reversal confirmed that the relative ordering of high-risk versus low-risk adenomas was insensitive to the choice of root assignment (S4 Fig in S1 File).

### In silico transcriptomic analysis identifies PTGS2 upregulation in an independent CRC cohort

**Fig 7:**
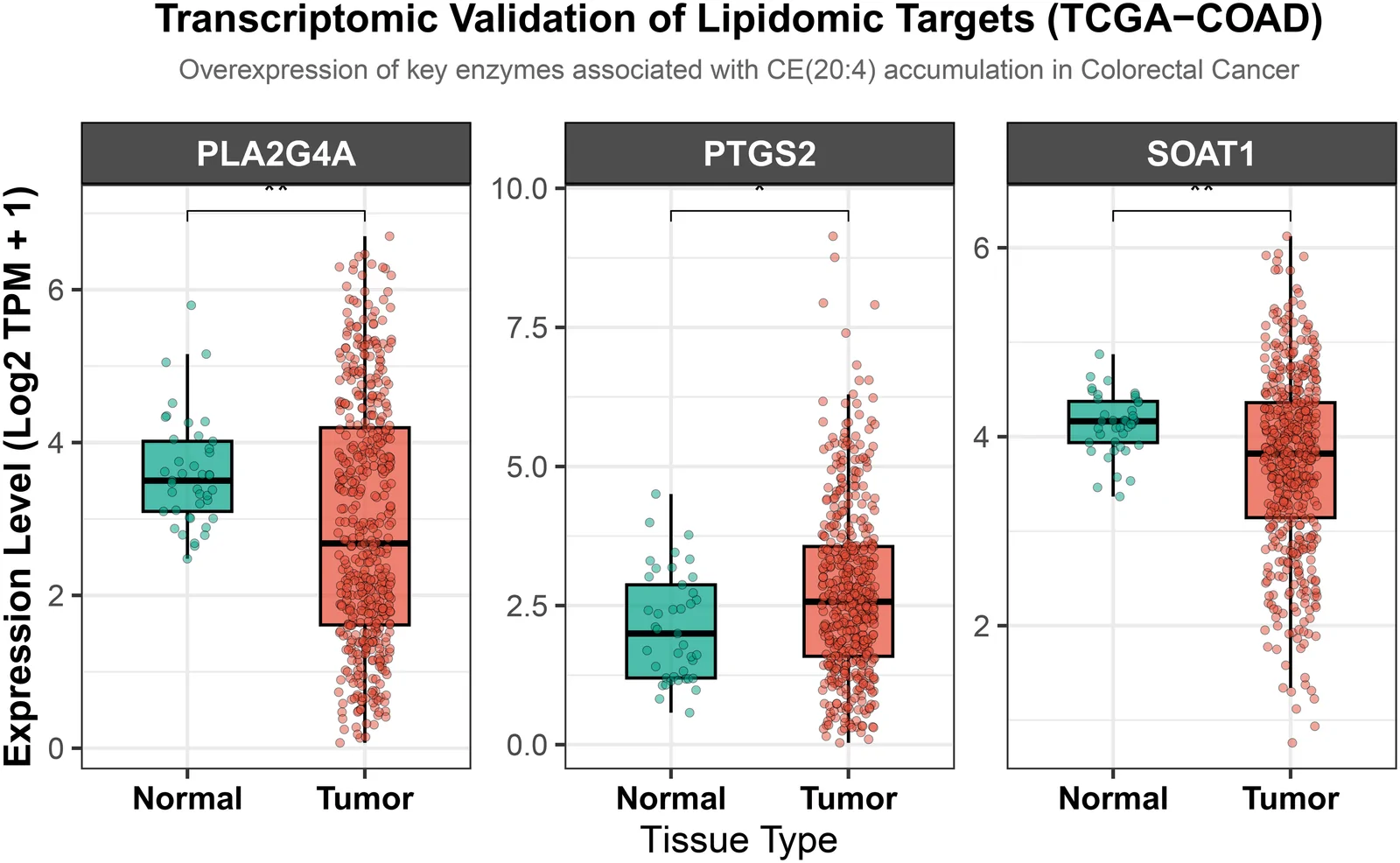
Dysregulation of key enzymes involved in CE(20:4) and arachidonic acid metabolism in the TCGA-COAD cohort. Normal tissue (*n* = 41) vs. primary tumor (*n* = 481). Boxplots show log2(TPM + 1) expression values. The center line denotes the median, box limits indicate the interquartile range (IQR), and whiskers extend to 1.5 × IQR. P-values from Welch’s t-test (confirmed by Wilcoxon rank-sum test; S10 Table in S2 File). *PTGS2* (COX-2): significantly upregulated ($\log_{2}\text{FC}=0.614$, *P* = 0.013); *SOAT1*: significantly downregulated ($\log_{2}\text{FC}=-0.402$, *P* = 0.002); *PLA2G4A*: significantly downregulated ($\log_{2}\text{FC}=-0.666$, *P* = 0.003). *PTGS2*, prostaglandin-endoperoxide synthase 2; *SOAT1*, sterol O-acyltransferase 1; *PLA2G4A*, phospholipase A2 group IVA.

Analysis of TCGA-COAD revealed significant upregulation of *PTGS2* (COX-2) in tumor tissue ($\log_{2}\text{FC}=0.614$, *P* = 0.013; Wilcoxon *P* = 0.0032, S10 Table in S2 File), while *SOAT1* and *PLA2G4A* were downregulated (Fig 7 and S1 Table in S2 File). Critically, these transcriptomic observations come from a cohort entirely independent of the fecal lipidomic discovery cohort; thus, the association between fecal CE(20:4) and tissue PTGS2 is a cross-cohort computational inference. Notably, the observed downregulation of SOAT1 (cholesterol ester synthase) and PLA2G4A (arachidonic acid release enzyme) alongside PTGS2 upregulation presents an apparent biochemical paradox: this expression profile would theoretically reduce CE(20:4) synthesis and AA release, yet fecal CE(20:4) accumulates. This discrepancy is examined in the Discussion.

### External single-molecule verification confirms CE(20:4) accumulation and an early adenoma-phase oxidative stress and eicosanoid perturbation

**Fig 8:**
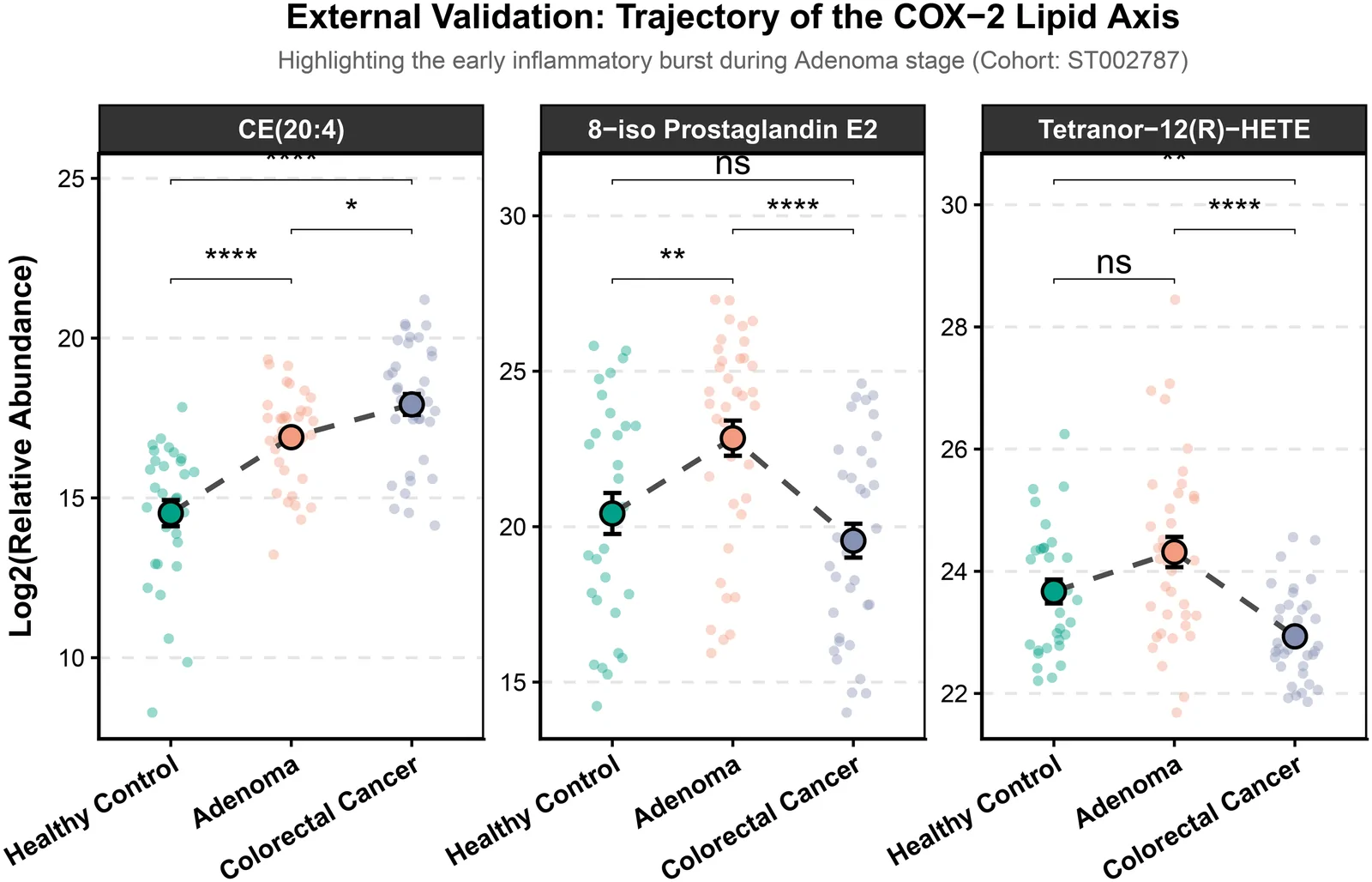
External single-molecule targeted verification of arachidonic acid-related lipid mediators across the healthy-adenoma-carcinoma continuum (ST002787, *n* = 102). Data are expressed as Log2 relative abundance ± standard error (SE). Between-group comparisons: Kruskal-Wallis test with Dunn’s post-hoc test and Benjamini-Hochberg correction (full results in S8 and S9 Tables in S2 File). (A) CE(20:4) (cholesteryl arachidonate): progressive increase from healthy to CRC; adenoma vs. CRC difference is not significant (*P* = 0.055), consistent with plateauing at the adenoma stage. (B) 8-iso Prostaglandin E2 (isoprostane; non-enzymatic lipid peroxidation product; oxidative stress marker): peaks in adenoma stage. (C) Tetranor-12(R)-HETE (12-HETE metabolite; primarily LOX pathway): peaks in adenoma stage. Significance levels: * *P* < 0.05, ** *P* < 0.01, *** *P* < 0.001, ns: not significant. Note: 8-iso Prostaglandin E2 and Tetranor-12(R)-HETE are not direct COX-2 enzymatic products.

In the independent ST002787 cohort, CE(20:4) showed a progressive increase across the healthy-adenoma-carcinoma continuum (univariate AUC for adenoma detection = 0.836, based on CE(20:4) alone). Full between-group comparisons are presented in S8 and S9 Tables in S2 File. Notably, CE(20:4) levels differed significantly between healthy controls and adenoma ($P=7.7\times 10^{-5}$) and between healthy controls and CRC ($P=1.8\times 10^{-8}$), but not between adenoma and CRC (*P* = 0.055), consistent with its proposed role as an early event marker that plateaus at the adenoma stage. Eicosanoid mediators associated with arachidonic acid metabolism, particularly 8-iso Prostaglandin E2 (an isoprostane marker of oxidative stress) and Tetranor-12(R)-HETE (a 12-HETE metabolite primarily from the LOX pathway), peaked during the adenoma stage (adjusted *P* < 0.01 vs. healthy controls; adenoma vs. CRC adjusted *P* < 0.001 for both; Fig 8). We note that 8-iso PGE2 is formed via non-enzymatic lipid peroxidation and is not a direct COX-2 enzymatic product; however, oxidative stress is known to be closely associated with COX-2 induction. This pattern is therefore consistent with an early oxidative stress and eicosanoid perturbation involvement but does not independently establish COX-2 pathway activation.

### Single-cell resolution shows stromal enrichment of PTGS2 expression

**Fig 9:**
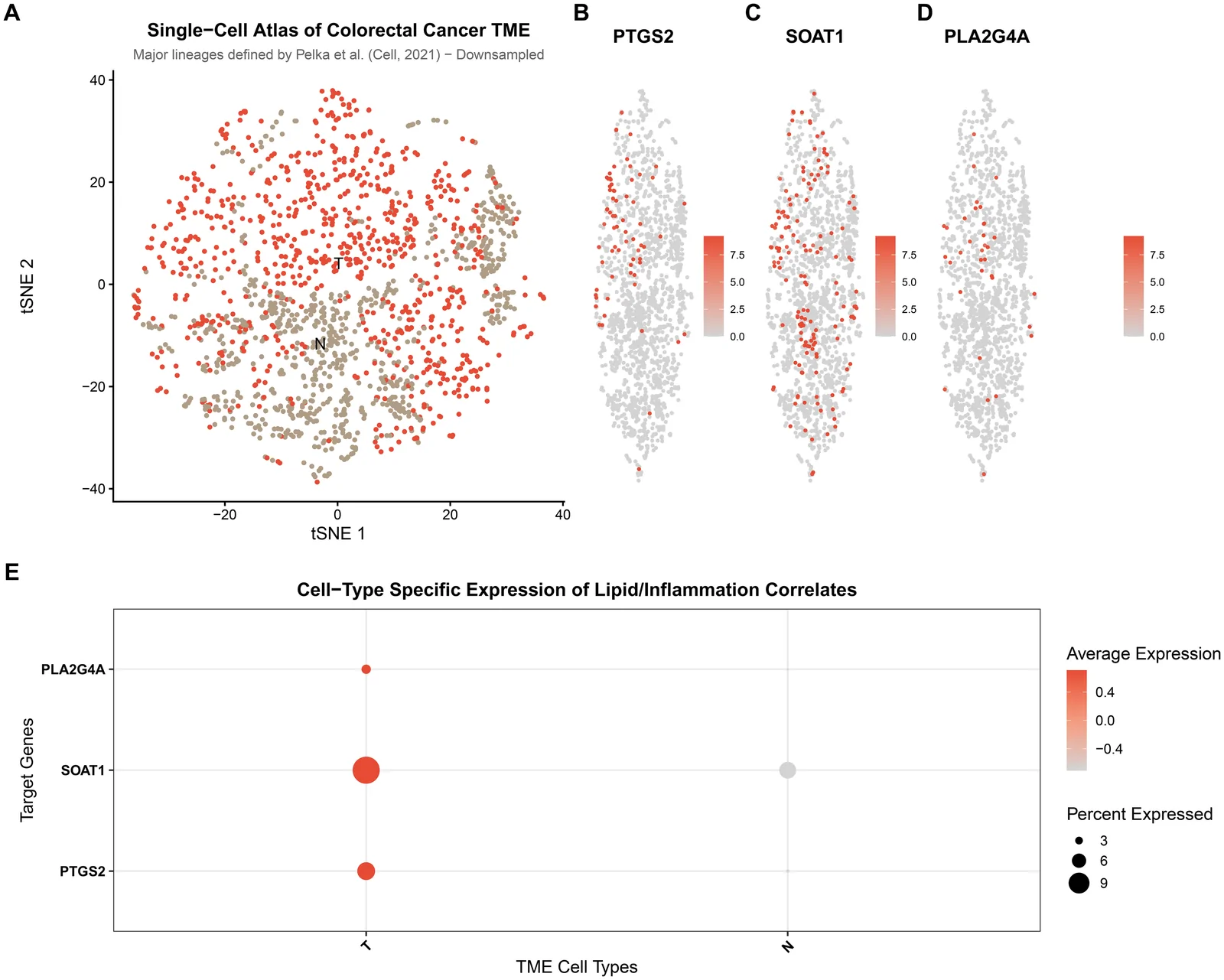
Single-cell RNA-seq analysis of the CRC tumor microenvironment (Broad Institute, c295). (A) tSNE embedding of single-cell transcriptomes, colored by cell type. (B) Feature plots showing *PTGS2* expression distribution across the tSNE space. (C) Dot plot illustrating *PTGS2* expression across cell types; dot size indicates the percentage of cells expressing *PTGS2*, and color intensity indicates mean expression level. *PTGS2* is predominantly enriched in tumor-associated macrophages (TAMs) and cancer-associated fibroblasts (CAFs) rather than in malignant epithelial cells. TPM, transcripts per million. These data are from fully developed CRC tissue, not adenomas.

In the CRC scRNA-seq atlas, consistent with previous reports, *PTGS2* was predominantly expressed in tumor-associated macrophages (TAMs) and cancer-associated fibroblasts (CAFs) rather than in malignant epithelial cells (Fig 9 and S2 Table in S2 File). It is important to note that this single-cell dataset is derived from fully developed CRC, not adenomas, and lacks paired lipidomic information; therefore, the cellular origin of fecal CE(20:4) cannot be directly inferred from these data.

## Discussion

### Principal findings and context

Using an integrative computational framework, we prioritized CE(20:4) as a candidate fecal lipidomic feature associated with a CRC-like metabolic state in colorectal adenomas. The prioritization of CE(20:4) was robust across multiple sensitivity analyses: it was the only feature consistently selected across two distinct imputation strategies (KNN and LOD/2; S7 Table in S2 File), and it achieved 100% bootstrap selection frequency (S11 Table in S2 File). The FL-MRS further revealed that a substantial subset of adenomas displays a CRC-like metabolic profile, suggesting hidden biological heterogeneity. However, every conclusion must be contextualized within the inherent constraints of our purely computational, multi-cohort design.

### The COX-2/CE(20:4) link: An in silico-generated hypothesis

Our SHAP-driven prioritization of CE(20:4) aligns with the known role of arachidonic acid metabolism in gastrointestinal inflammation [29]. Classical studies have shown that COX-2 is upregulated in a substantial proportion of colorectal adenomas [30], and that APC mutation-driven COX-2 induction is an early event in intestinal tumorigenesis [31]. The concurrent peak of eicosanoid mediators during the adenoma stage therefore provides circumstantial support for an early inflammatory and oxidative stress involvement. However, several important caveats must be addressed. First, 8-iso Prostaglandin E2 is an isoprostane formed via non-enzymatic lipid peroxidation rather than COX-2-specific enzymatic activity; therefore, the external verification data demonstrate elevated oxidative stress during the adenoma stage, not COX-2 pathway activation per se. The association between oxidative stress and COX-2 induction is well-established in colorectal carcinogenesis, but the two should not be conflated. Second, the observed downregulation of SOAT1 and PLA2G4A in TCGA data presents an apparent biochemical paradox: if the cholesterol ester synthase (SOAT1) and the AA-releasing enzyme (PLA2G4A) are both suppressed, the accumulation of CE(20:4) in feces cannot be readily explained by the canonical host-tissue pathway.

We propose the following non-mutually-exclusive hypotheses, distinguished by disease stage. For the early adenoma-phase accumulation of CE(20:4): (1) inflammatory signals from the adenoma microenvironment, potentially involving stromal-derived COX-2 activity, may drive local lipid remodeling and cholesterol esterification in pre-malignant epithelial cells prior to detectable transcriptional changes. The mechanistic link between stromal COX-2-driven inflammation and epithelial CE(20:4) accumulation remains experimentally unvalidated; it may involve indirect upregulation of cholesterol esterification enzymes via downstream inflammatory signaling cascades, rather than direct enzymatic synthesis by COX-2. (2) Gut microbiota possess their own cholesterol esterification machinery and may contribute substantially to the luminal CE(20:4) pool, a possibility that cannot be evaluated without paired metagenomic data. The observation that CE(20:4) levels did not differ significantly between adenoma and CRC (*P* = 0.055) suggests that the elevation may plateau at the adenoma stage, although this interpretation requires larger longitudinal studies. For the sustained elevation during the CRC stage: (3) exfoliated tumor cells undergoing necrosis may release pre-formed cholesteryl esters from intracellular lipid droplets directly into the gut lumen, bypassing the need for active SOAT1-mediated synthesis and providing an alternative mechanism for CE(20:4) accumulation that is independent of the canonical host-tissue pathway. The static transcriptomic snapshot from TCGA, showing SOAT1 and PLA2G4A downregulation alongside PTGS2 upregulation, may represent a compensatory state in established CRC rather than reflecting the dynamic processes during adenoma-carcinoma transition.

To move beyond the correlative nature of the current findings, future studies should: (1) quantify CE(20:4) in matched fecal, mucosal, and plasma samples collected prospectively from patients with adenomas of varying histological risk; (2) apply spatial lipidomics to determine whether CE(20:4) localizes to epithelial, stromal, or immune compartments; (3) use adenoma-derived organoid models to test whether COX-2 inhibition or SOAT1 modulation alters CE(20:4) abundance and downstream eicosanoid profiles. Such studies will be essential to determine the true origin and temporal dynamics of luminal CE(20:4).

### Methodological considerations and sensitivity analyses

Our computational pipeline incorporated several methodological choices that warrant careful discussion. First, the feature overlap between the discovery (ST003798) and external verification (ST002787) cohorts was limited to 1 of 11 features (S6 Table in S2 File), precluding independent replication of the full FL-MRS model. This limited overlap is attributable to inherent technical differences between the two cohorts, including distinct mass spectrometry platforms, chromatographic separation methods, and untargeted feature extraction and alignment pipelines. Such cross-platform variability is a well-recognized challenge in untargeted lipidomics and underscores the need for standardized data acquisition and processing protocols in future multi-cohort studies.

Second, our pseudotime trajectory inference was based on cross-sectional data using the first two principal components. While root node reversal confirmed that the relative sample ordering was insensitive to this choice (S4 Fig in S1 File), the inferred trajectory should not be equated with true longitudinal disease progression.

Third, the substantial divergence between feature sets selected under different imputation strategies (Jaccard = 0.059; S7 Table in S2 File) and the variable bootstrap selection frequencies (S11 Table in S2 File) highlight the inherent sensitivity of biomarker discovery pipelines to preprocessing decisions. This instability is a well-documented challenge in metabolomics and lipidomics, where data are sparse and affected by left-censoring. It also underscores the importance of transparent reporting of all preprocessing choices and the need for external verification. Notably, this analytical instability provides a methodological explanation for the limited overlap of lipidomic biomarkers reported across independent fecal CRC metabolomics studies. The observation that CE(20:4) remained robust across all sensitivity analyses, while the broader feature set was unstable, suggests that focusing on individual robustly-prioritized molecules may be a more reliable strategy than relying on multi-feature composite scores in untargeted lipidomics biomarker research.

Fourth, an important implication of the observed feature instability concerns the FL-MRS-defined adenoma high-risk proportion. The finding that most model features are sensitive to preprocessing choices implies that the specific 51.7% figure would vary under alternative analytical pipelines. We therefore emphasize that this proportion should not be interpreted as a stable estimate of adenoma metabolic heterogeneity; rather, it serves as a qualitative illustration that substantial heterogeneity exists. The qualitative conclusion—that a subset of adenomas exhibits a CRC-like metabolic profile—is robust, as it is supported by consistent qualitative trends across FL-MRS, SHAP analysis, exploratory pseudotime trajectory, and the external verification of CE(20:4). Future studies employing standardized preprocessing protocols and independent validation cohorts are needed to establish a more precise estimate of the metabolically defined high-risk adenoma prevalence.

Finally, the extreme-phenotype strategy used here is not specific to colorectal cancer. It is well-suited to any disease continuum in which clinically defined intermediate stages are heterogeneous, because it anchors the model on unambiguous extremes and then interrogates the intermediate group without imposing potentially arbitrary subclass labels. Examples include early cognitive decline, subclinical cardiovascular remodeling, and inflammatory bowel disease-associated dysplasia. This generalizability further supports the methodological value of the framework beyond the specific biological findings of this study.

### Microenvironmental context and cellular sources

Our re-analysis of public scRNA-seq data, consistent with previously reported CRC microenvironment features, confirms that *PTGS2* is enriched in TAMs and CAFs. While this pattern is intriguing, it is derived from CRC samples and does not directly inform on the adenoma-stage microenvironment. The hypothesis that stromal cells contribute to the fecal lipidomic shift should be tested in future studies that combine fecal lipidomics with tissue-resolved transcriptomics or imaging mass spectrometry from the same individuals.

### The gut microbiome as an unmeasured contributor

A significant limitation of this study is the absence of paired metagenomic data. The fecal lipidome is a product of both host and microbial metabolism, and cholesterol esters such as CE(20:4) may be produced, modified, or degraded by specific gut bacteria. Several bacterial taxa implicated in colorectal carcinogenesis, including *Fusobacterium nucleatum* and enterotoxigenic *Bacteroides fragilis*, are known to modulate host inflammatory and lipid signaling pathways. Furthermore, bacterial cholesterol esterification activity has been documented in the gut microbiome. The observed CE(20:4) accumulation may therefore partially reflect microbial community alterations rather than, or in addition to, host-tissue metabolic reprogramming.

In addition to direct lipid metabolism, gut microbial communities may influence fecal CE(20:4) through indirect mechanisms. Microbial-derived uremic toxins, including indoxyl sulfate and p-cresyl sulfate, have been linked to chronic inflammation, oxidative stress, and colorectal cancer progression [32]. This is particularly relevant to our observation of elevated 8-iso Prostaglandin E2—a non-enzymatic lipid peroxidation product—in the adenoma stage. This suggests that early microbial dysbiosis may contribute to the oxidative stress phenotype captured by our lipidomic analysis. Future prospective studies should incorporate shotgun metagenomics or metatranscriptomics to disentangle host-derived from microbially-derived lipid signals and to assess whether specific microbial taxa contribute to the FL-MRS-defined high-risk metabolic phenotype.

### Clinical implications remain speculative

The FL-MRS cutoff of 0.446, validated through bootstrap methods, provides a computational tool for exploring metabolic heterogeneity among adenomas. The well-established chemopreventive benefit of NSAIDs [33–35] aligns with our observation of early inflammatory and oxidative stress involvement, supporting the biological plausibility of our findings. However, the present study lacks the prospective design, clinical metadata, and histopathological correlates necessary to evaluate FL-MRS as a risk stratification tool. As discussed above, the 51.7% proportion substantially exceeds the known clinical adenoma-carcinoma progression rate, confirming that FL-MRS should not be misinterpreted as a predictor of malignant transformation. Rather, it provides a non-invasive, data-driven metric for quantifying the degree to which an adenoma’s metabolic profile resembles that of CRC. This may serve as a starting point for future studies investigating whether this metabolic similarity correlates with clinical outcomes. FL-MRS is not proposed as a diagnostic or risk-prediction tool; its intended use is as a research instrument for selecting adenoma patients for mechanistic studies or chemoprevention trials. Factors such as BMI, dietary habits, statin use, and undocumented NSAID intake could confound both the lipidomic profiles and the apparent heterogeneity among adenomas. Therefore, our results do not support immediate clinical translation.

### Study limitations and generalizability

This study has several critical limitations that must be explicitly acknowledged. First, it is a purely computational re-analysis of public data without independent experimental validation; all findings are hypothesis-generating. Second, the fecal lipidomic, tissue transcriptomic, and single-cell data originate from independent, non-matched cohorts, making all cross-omic associations inferential rather than causal. Third, external verification was limited to single-molecule targeted trend verification of CE(20:4) and two eicosanoid mediators, as full model replication was not feasible due to minimal inter-cohort feature overlap (S6 Table in S2 File). Fourth, the absence of key clinical covariates (age, sex, BMI, medication history, dietary habits, NSAID/statin exposure) in public datasets prevents adjustment for potential confounders and limits the interpretation of adenoma heterogeneity. The lack of adenoma histopathological grading precludes validation of FL-MRS against established histological risk criteria. Fifth, the analyzed cohorts are predominantly of Western ancestry, and generalizability to other populations is unknown. Sixth, the cross-sectional pseudotime analysis is a computational ordering that cannot substitute for true longitudinal sampling. Seventh, our sensitivity analysis revealed that the choice of missing value imputation strategy substantially affected the composition of the LASSO-selected feature set (S7 Table in S2 File), and bootstrap stability analysis confirmed variable selection frequencies for most features (S11 Table in S2 File), highlighting an inherent challenge in biomarker discovery from untargeted lipidomics. Eighth, the absence of metagenomic data precludes assessment of microbial contributions to the fecal lipidomic profiles. Ninth, fecal CE(20:4) levels are directly influenced by dietary intake of cholesterol and arachidonic acid, as well as by BMI, medication use, and gut microbiota composition, which could not be controlled for in this analysis of public data. Tenth, the FL-MRS cutoff of 0.446 was derived from Youden’s index applied to the healthy-versus-CRC binary classification and has not been calibrated against any adenoma-specific gold standard; its application to the adenoma population therefore represents an extrapolation that requires prospective validation with clinical endpoints. Future prospective studies with matched biospecimens, comprehensive clinical annotation, standardized lipidomics platforms, metagenomic profiling, and experimental validation are essential to confirm or refute the hypotheses generated here.

## Conclusions

Through an integrative computational framework applied to publicly available fecal lipidomic and transcriptomic datasets, we generated the hypothesis that CE(20:4) and the COX-2 inflammatory pathway may represent candidate features of a CRC-like metabolic state in colorectal adenomas. The prioritization of CE(20:4) was robust to alternative preprocessing strategies and achieved 100% bootstrap selection frequency, while the broader 11-feature signature showed variable stability. The FL-MRS revealed substantial metabolic heterogeneity among adenomas, serving as a computational tool for quantifying CRC-like metabolic similarity, though its clinical relevance remains unvalidated. FL-MRS is not proposed as a clinical diagnostic or risk-prediction tool; rather, it serves as a research instrument for guiding future validation studies. Beyond the specific biological hypothesis, this study provides a transparent, reproducible analytical framework for secondary use of public omics data—integrating extreme-phenotype modeling, multi-dimensional sensitivity analyses, and explainable AI—that can be adapted to investigate disease progression trajectories in other clinical contexts where intermediate disease states are difficult to characterize. We have highlighted several critical areas for future investigation, including the resolution of the apparent biochemical paradox in CE(20:4) accumulation, the potential contribution of the gut microbiome, and the need for matched tissue-fecal paired prospective cohorts. All code and data sources have been made publicly available to ensure reproducibility and to facilitate the independent prospective and experimental studies needed to test these hypotheses.

## Supporting information

S1 File Supplementary Figures S1-S4. This file contains Supplementary Figures S1-S4. S1 Fig: Model calibration and decision curve analysis. S2 Fig: Study design flowchart. S3 Fig: Out-of-bag error convergence plot. S4 Fig: Pseudotime root node sensitivity analysis.
(PDF)

S2 File Supplementary Tables S1-S11. This file contains Supplementary Tables S1-S11 in a single Excel workbook. S1 Table: TCGA-COAD differential expression statistics. S2 Table: Single-cell expression metrics. S3 Table: Detailed diagnostic performance metrics. S4 Table: Feature coefficient and importance scores. S5 Table: Multi-model comparison. S6 Table: Feature overlap between cohorts. S7 Table: Sensitivity analysis of missing value imputation. S8 Table: Kruskal-Wallis test results. S9 Table: Dunn’s post-hoc comparisons. S10 Table: Wilcoxon rank-sum validation. S11 Table: LASSO bootstrap stability analysis.
(XLSX)

S3 File TRIPOD Reporting Checklist. Completed TRIPOD checklist for transparent reporting of a multivariable prediction model study.
(DOCX)
