# Supplementary material for: Integrative computational analysis of public fecal lipidomics and transcriptomics datasets suggests a candidate association between the COX-2 pathway and CE(20:4) in colorectal adenoma-carcinoma progression
Source: PLoS One. 2026 Sep 25;21(9):e0358259. doi: 10.1371/journal.pone.0358259 (PMC13614635; doi:10.1371/journal.pone.0358259)
Supplement: Supplementary file 1 [file pone.0358259.s001.pdf]

Supplementary Materials: Integrative Computational Analysis of Public Fecal Lipidomics and Transcriptomics Datasets

Bing Tang, Yanru Chen, Xia Yang, Hanlin Gong

Supplementary Figures

Supplementary Figure S1: Model calibration and decision curve analysis

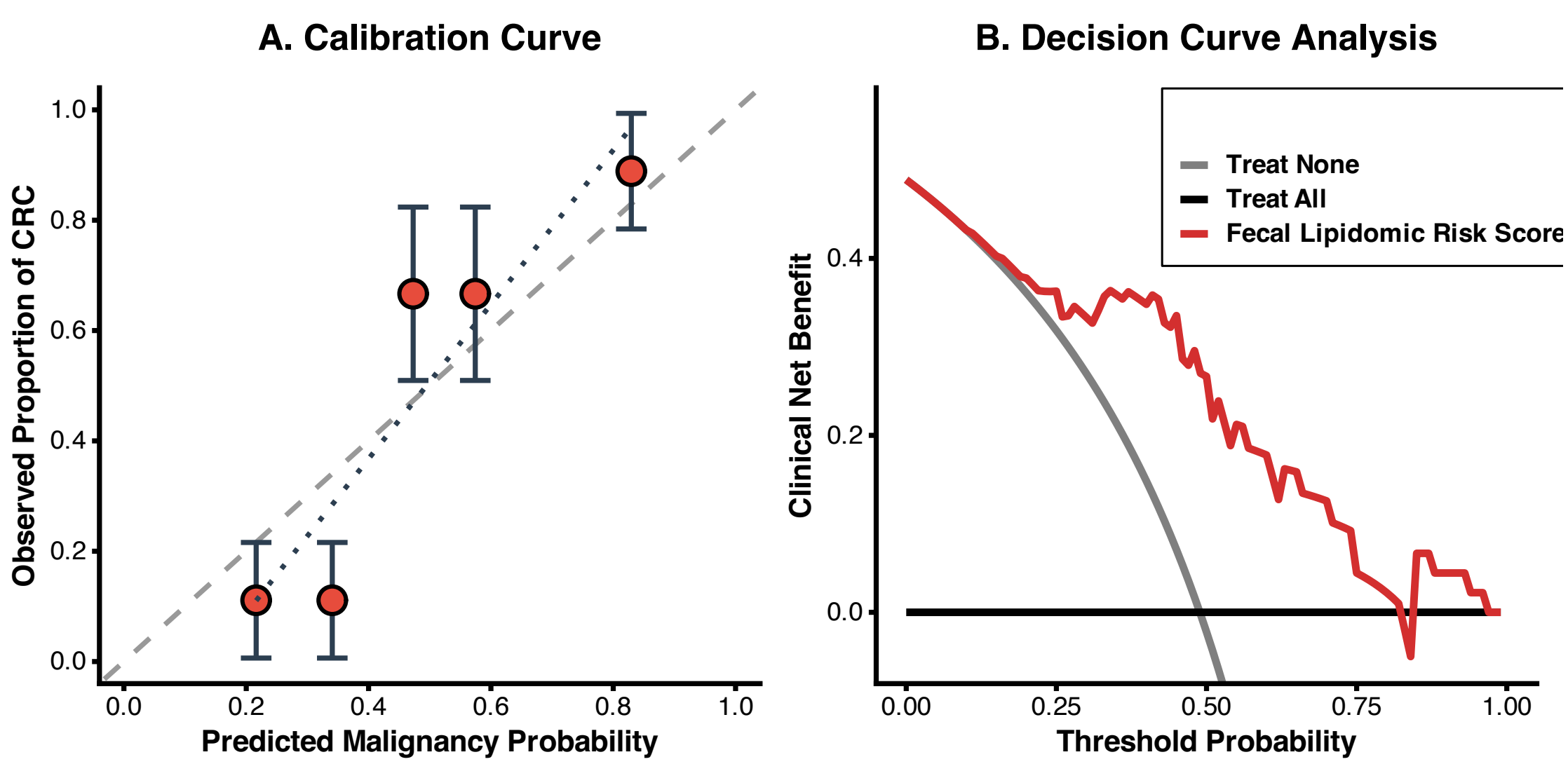

Figure 1: Calibration curve and decision curve analysis (DCA) for the Fecal Lipidomic Malignancy Risk Score (FL-MRS). (A) Calibration curve comparing predicted risk against observed CRC proportions. (B) DCA demonstrating net clinical benefit across a range of threshold probabilities.

Supplementary Figure S2: Study design flowchart

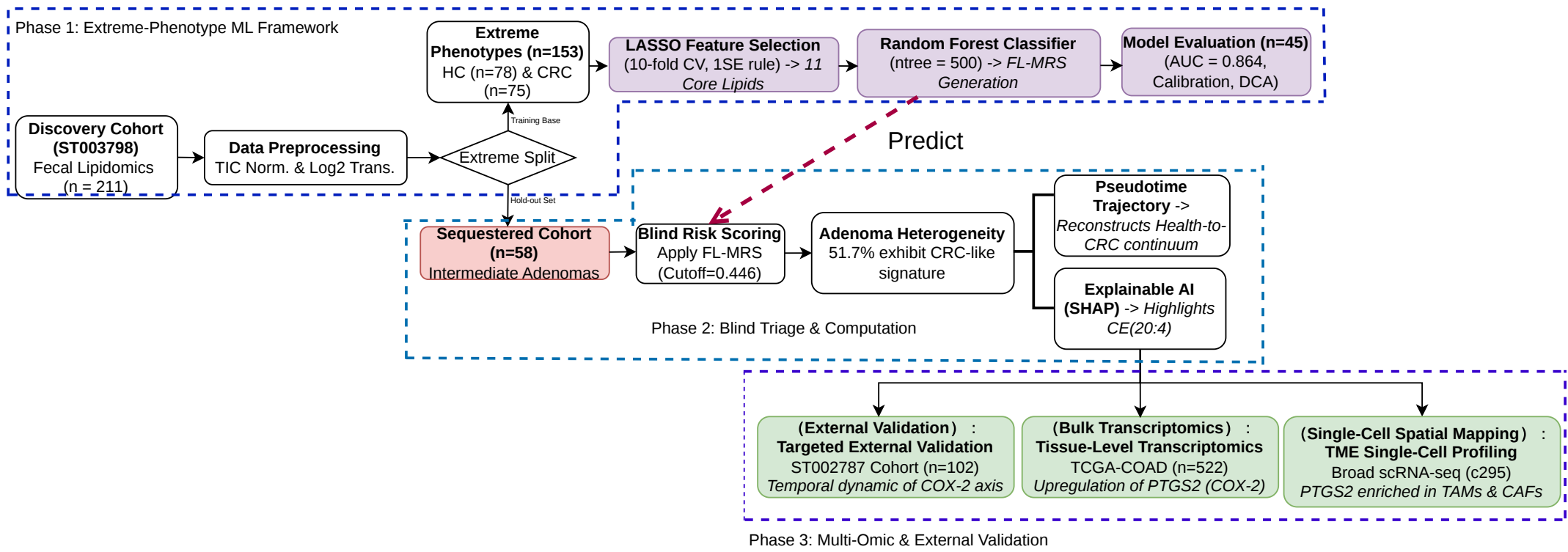

Figure 2: Schematic overview of the multi-cohort study design. Datasets used include ST003798 (discovery fecal lipidomics), ST002787 (external verification), TCGA-COAD (bulk transcriptomics), and Broad Institute c295 (single-cell RNA-seq).

Supplementary Figure S3: Random Forest out-of-bag error convergence

Supplementary Figure S4: Pseudotime root node sensitivity analysis

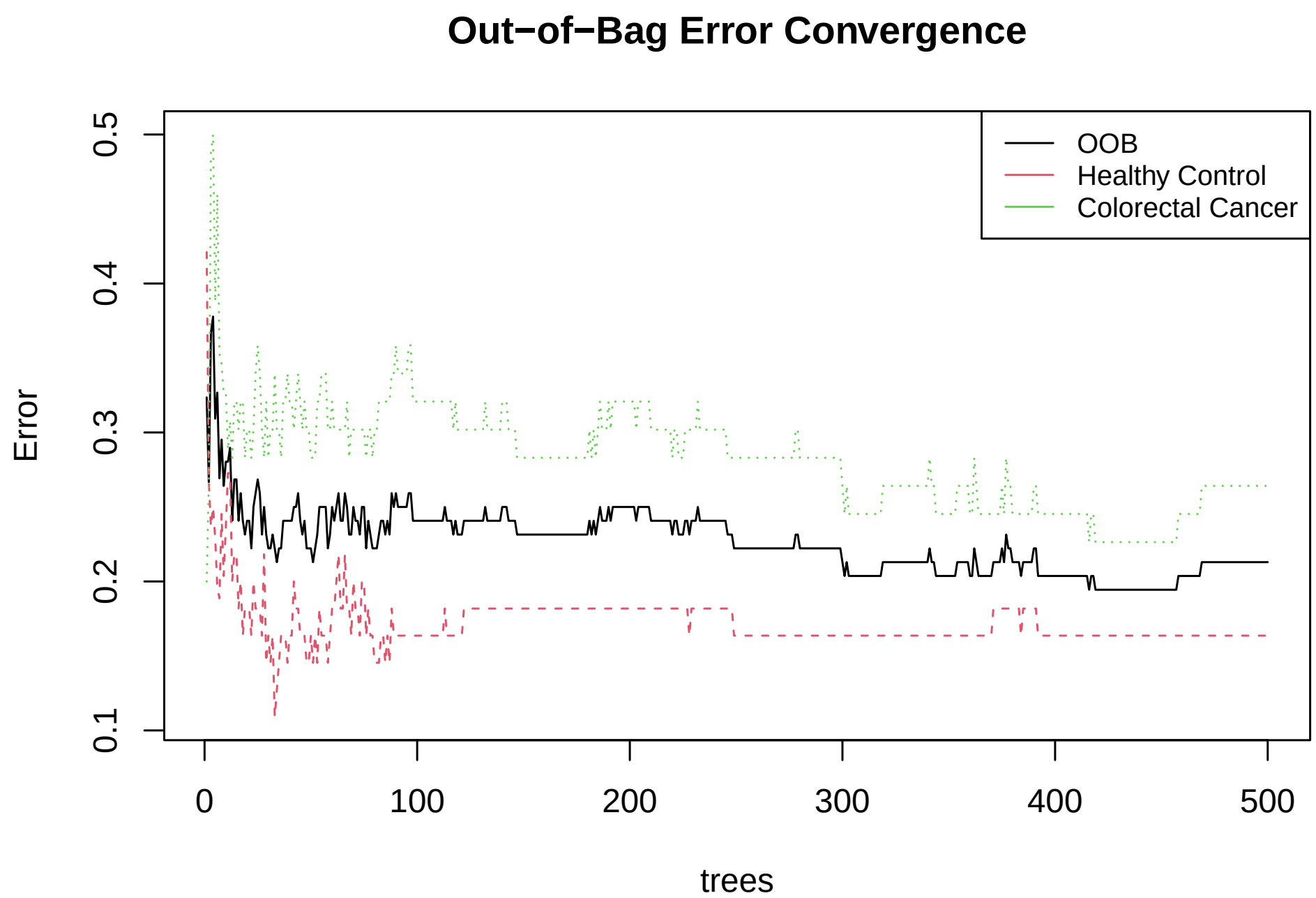

Figure 3: Out-of-bag (OOB) error as a function of the number of trees in the Random Forest model. The OOB error stabilized at approximately 416 trees with a minimum error rate of 0.1944, confirming that the chosen $ntree = 500$ was sufficient for convergence.

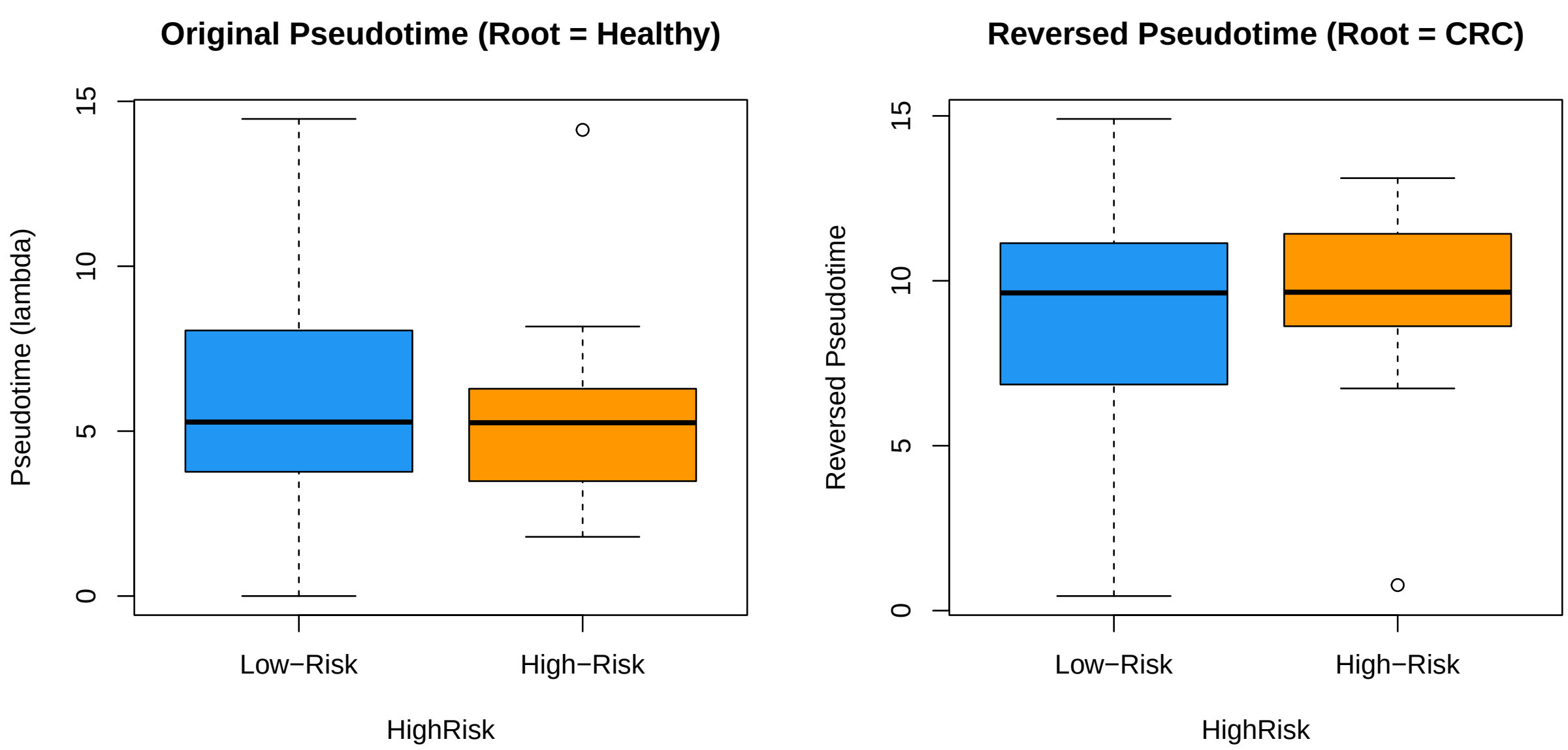

Figure 4: Sensitivity analysis of pseudotime trajectory to root node assignment. (A) Original pseudotime with healthy controls designated as the root. (B) Reversed pseudotime with CRC designated as the root. In both panels, high-risk adenomas are shifted toward the CRC terminus relative to low-risk adenomas, confirming that the relative ordering is insensitive to root node choice ($P = 0.6631$ for both comparisons, Wilcoxon rank-sum test).
